# Enhanced monocyte recruitment and delayed alternative macrophage polarization accompanies impaired repair following myocardial infarction in C57BL/6 compared to BALB/c mice

**DOI:** 10.1111/cei.13330

**Published:** 2019-06-17

**Authors:** I. S. Toor, D. Rückerl, I. Mair, A. Thomson, A. G. Rossi, D. E. Newby, J. E. Allen, G. A. Gray

**Affiliations:** ^1^ BHF/University Centre for Cardiovascular Science, Queen’s Medical Research Institute, University of Edinburgh Edinburgh UK; ^2^ Faculty of Biology, Medicine and Health, School of Biological Sciences University of Manchester Edinburgh UK; ^3^ MRC Centre for Inflammation Research, Queen’s Medical Research Institute, University of Edinburgh Edinburgh UK

**Keywords:** C57BL/6, BALB/c, macrophage polarization, monocyte, myocardial infarction

## Abstract

Activation of the innate immune response following myocardial infarction (MI) is essential for infarct repair. Preclinical models of MI commonly use C57BL/6 mice, which have a type 1‐dominant immune response, whereas other mouse strains such as BALB/c mice have a type 2‐dominant immune response. We compared C57BL/6 and BALB/c mice to investigate whether predisposition towards a proinflammatory phenotype influences the dynamics of the innate immune response to MI and associated infarct healing and the risk of cardiac rupture. MI was induced by permanent coronary artery ligation in 12–15‐week‐old male wild‐type BALB/c and C57BL/6 mice. Prior to MI, C57BL/6 mice had a lower proportion of CD206^+^ anti‐inflammatory macrophages in the heart and an expanded blood pool of proinflammatory Ly6C^high^ monocytes in comparison to BALB/c mice. The systemic inflammatory response in C57BL/6 mice following MI was more pronounced, with greater peripheral blood Ly6C^high^ monocytosis, splenic Ly6C^high^ monocyte mobilization and myeloid cell infiltration of pericardial adipose tissue. This led to an increased and prolonged macrophage accumulation, as well as delayed transition towards anti‐inflammatory macrophage polarization in the infarct zone and surrounding tissues of C57BL/6 mice. These findings accompanied a higher rate of mortality due to cardiac rupture in C57BL/6 mice compared with BALB/c mice. We conclude that lower post‐MI survival of C57BL/6 mice over BALB/c mice is mediated in part by a more pronounced and prolonged inflammatory response. Outcomes in BALB/c mice highlight the therapeutic potential of modulating resolution of the innate immune response following MI for the benefit of successful infarct healing.

## Introduction

Myocardial infarction (MI) is the most common cause of cardiovascular mortality. ST elevation MI (STEMI), the most serious form, occurs following acute thrombotic occlusion of a coronary artery leading to interruption of blood flow to the myocardium 1. The focus of treatment following STEMI is to limit the size of myocardial injury with the rapid restoration of coronary artery patency using emergency percutaneous coronary intervention 1, 2. In response to myocardial injury, there is activation of the innate immune response resulting in the recruitment of neutrophils and monocytes to the infarct zone 3. While myeloid cells are required for successful infarct repair, an excessive and prolonged innate immune response can lead to infarct expansion and impaired healing 3, 4, 5. Severe post‐infarct inflammation is associated with cardiac rupture 6, which is a catastrophic complication of acute MI with an in‐hospital mortality of 60–90% for left ventricular free wall rupture 7. The incidence of rupture is between 1 and 3% in acute MI patients 7 and occurs 2–6 days post‐MI 8. This is a similar time‐frame to that seen in mice, which are the only mammalian species used for MI research that develops cardiac rupture following a transmural MI 8.

Variation in the predisposition to cardiac rupture between mouse strains has been attributed to differences in a number of factors, including blood pressure 9, availability of matrix metalloproteinases 10 and extracellular matrix synthesis 11. The magnitude of CD45^+^ leucocyte recruitment 12 and expression of proinflammatory cytokines and chemokines 13 in the infarcted myocardium also varies between mouse strains.

Preclinical mouse models of MI commonly use proinflammatory type 1 immune response dominant C57BL/6 mice, which have a higher rate of cardiac rupture than the more anti‐inflammatory type 2 immune response dominant BALB/c mice 7, 9, 14. The T helper type 1 (Th1)/Th2 paradigm of immunological development was initially established in *Leishmania major* infection 15. A lack of early interleukin (IL)‐12 production in BALB/c mice results in an IL‐4‐driven anti‐inflammatory Th2 immune response, while the IL‐12‐driven proinflammatory Th1 immune response of C57BL/6 mice is characterized by the production of interferon (IFN)‐γ 16, 17, 18. Macrophage function is one of the key mechanisms that shape this characteristic immune response of C57BL/6 and BALB/c mice 19. The macrophage phenotype also determines the resolution of inflammation, which is marked by the transition from a proinflammatory to an anti‐inflammatory macrophage phenotype 20. This transition has a central role in infarct healing, as failure of macrophages to acquire an anti‐inflammatory phenotype following MI is associated with an increased rate of cardiac rupture 21.

The role of the immune response is increasingly being explored as a therapeutic target in a wide range of diseases. It is important to understand whether predisposition towards a proinflammatory phenotype influences the dynamics of myeloid cell recruitment and macrophage phenotype following induction of MI and its correlation with infarct healing and the risk of cardiac rupture. We compared C57BL/6 and BALB/c mice with respect to monocyte mobilization and macrophage polarization, as well as the temporal and spatial pattern of the innate immune response following MI.

## Methods

### Animals

All mice were kept under specific pathogen‐free conditions. Ten to 12‐week‐old wild‐type (WT) male BALB/c and C57BL/6 mice were purchased from Harlan Laboratories (Loughborough, UK) and maintained at the University of Edinburgh for at least 2 weeks to acclimatize them to a 12‐h/12‐h light/dark cycle, with free access to standard diet and water prior to surgery. All experiments were conducted under a Project Licence granted by the UK Home Office and approved by the University of Edinburgh Animal Welfare and Ethical Review Body.

### Infarct model

The mouse MI model was induced by permanent ligation of the left coronary artery, as previously described 22. In brief, mice were anaesthetized with 2% isoflurane, intubated and mechanically ventilated (120 strokes/min, 200 μl stroke volume, Hugo Sachs Elektronik Minivent, Cambridge, UK). After a left‐sided thoracotomy, MI was induced by ligating the proximal portion of the left coronary artery using an 8‐0 PROLENE suture (Ethicon, Somerville, NJ, USA). The chest was then closed in layers and the pneumothorax was evacuated. Buprenorphine (0·05 mg/kg) was given subcutaneously for perioperative analgesia, in addition to 1 ml 0.9% saline for rehydration. Twenty‐four hours after performing coronary artery ligation, a tail blood sample (30 μl) was collected (in 3·2% citrate buffer) for assay of troponin I by enzyme‐linked immunosorbent assay (ELISA) [Life Diagnostics (West Chester, PA, USA) high sensitivity mouse cardiac troponin‐I ELISA kit, according to the manufacturer’s instructions] to assess the size of injury.

### Cardiac imaging

Left ventricular structure and function was assessed at day 7 following MI by high‐resolution ultrasound (VisualSonics Vevo 770, Toronto, Canada) with a 707B 30 MHz ultrasound probe, as previously described 22. Mice were maintained under light anaesthesia with 2% isoflurane and with spontaneous respiration. Two‐dimensional images were obtained of parasternal long‐axis views of the left ventricle. Images were saved and analysed offline using the Vevo 770 high‐resolution imaging system, version 2.2.0 to calculate the left ventricular end‐diastolic area, left ventricular end‐systolic area and ejection fraction 22. The image analysis was performed in a single‐blinded manner.

### Histology

Mouse hearts were embedded in paraffin and 5‐µm sections cut using a microtome (Leica, Wetzlar, Germany) at three different levels below the level of coronary artery ligation. Scar size was assessed using Masson’s trichrome staining. The epicardial length of the infarct was selected manually and expressed as a percentage of the LV epicardial circumference.

### Flow cytometry

At 0, 1, 2, 4 and 10 days after MI, tail blood was taken and then animals were killed prior to harvesting the heart, pericardial adipose tissue and spleen. Immunofluorescence staining was performed on tail blood samples and single cell suspensions from heart digests, pericardial adipose tissue and spleens (Supporting information, Methods). Flow cytometric analysis was performed on an LSR II instrument (BD Biosciences, San Jose, CA, USA) and fluorochrome conjugated antibodies were used to define cell populations of interest using FlowJo software (Tree Star, Ashland, OR, USA) (Supporting information, Fig. S1). Results for the heart digests are expressed as cell number per infarct zone or remote zone, and total counts were calculated for spleens, pericardial adipose tissue and the peripheral blood.

### Statistical analysis

All data are expressed as mean ± standard error of mean. Comparisons between BALB/c mice *versus* C57BL/6 mice and ‘no MI *versus* time‐point’ for BALB/c and C57BL/6 mice were performed with the Wilcoxon rank‐sum test. Survival distributions were plotted using the Kaplan–Meier method and compared by the log‐rank test. The level of significance was set at *P* < 0·05. All statistical analyses were performed with spss version 15.0 statistical software package (SPSS Inc., Chicago, IL, USA).

## Results

### C57BL/6 mice have a higher rate of cardiac rupture than BALB/c mice following MI

**Figure 1:**
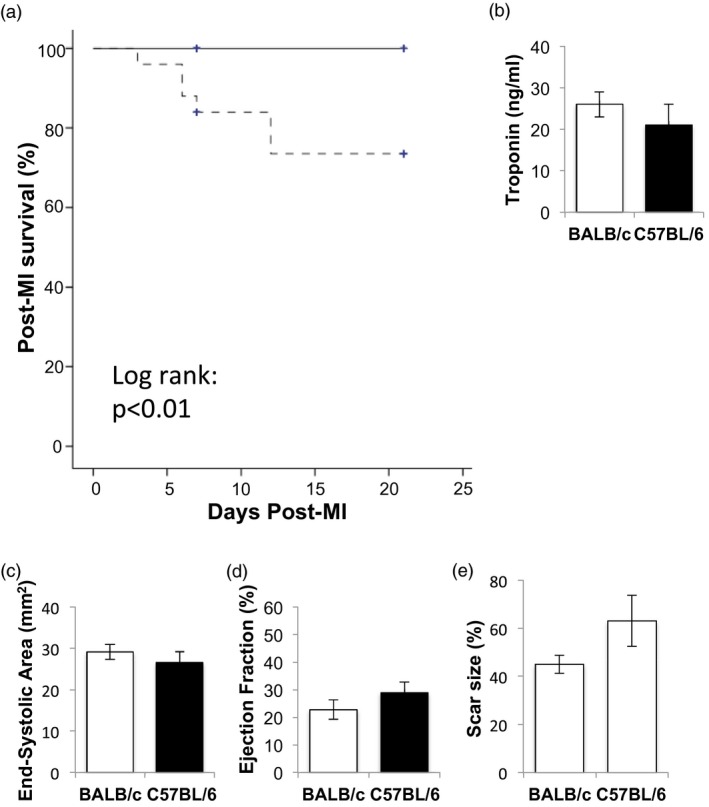
C57BL/6 mice have increased mortality due to cardiac rupture following myocardial infarction (MI). (a) Post‐MI survival in WT BALB/c (*n* = 19, solid line) and C57BL/6 (*n* = 17, dotted line) mice. (b) Troponin I concentration in blood collected at 24 h following induction of MI in C57BL/6 and BALB/c mice undergoing cardiac ultrasound at day 7 post‐MI (*n* = 7–10/group). (c) End‐systolic area at day 7 following MI. (d) Ejection fraction at day 7 following MI (*n* = 7–10/group). (e) Scar size expressed as a percentage of the left ventricle in BALB/c and C57BL/6 mice at day 7 post‐MI (*n* = 3–5/group).

C57BL/6 and BALB/c mice were monitored following induction of MI to determine the role of genetic background in cardiac repair. Plasma troponin I concentration in blood collected at 24 h following induction of MI by coronary artery ligation was comparable in C57BL/6 and BALB/c mice (Fig. 1a), indicating a similar extent of injury. However, the rate of mortality was higher in C57BL/6 mice (log‐rank *P* = 0·03, Fig. 1b). The deaths following infarction were all as a consequence of cardiac rupture, as diagnosed by massive blood loss in the thoracic cavity on postmortem examination 9, and 80% occurred between days 3 and 7 following MI.

Cardiac function was assessed 7 days after MI. High‐resolution ultrasound indicated that C57BL/6 and BALB/c mice had similar left ventricular size (Fig. 1c, end‐systolic left ventricular area, *P* = 0·27) and left ventricular function (Fig. 1d, left ventricular ejection fraction, *P* = 0·32; Supporting information, Table S1). Compared with BALB/c mice, scar size tended to be higher in C57BL/6 mice surviving at day 7 post‐MI, but this did not reach statistical significance (*P* = 0·22; Fig. 1e).

### Greater peripheral blood Ly6C^high^ monocytosis in C57BL/6 mice following MI

**Figure 2:**
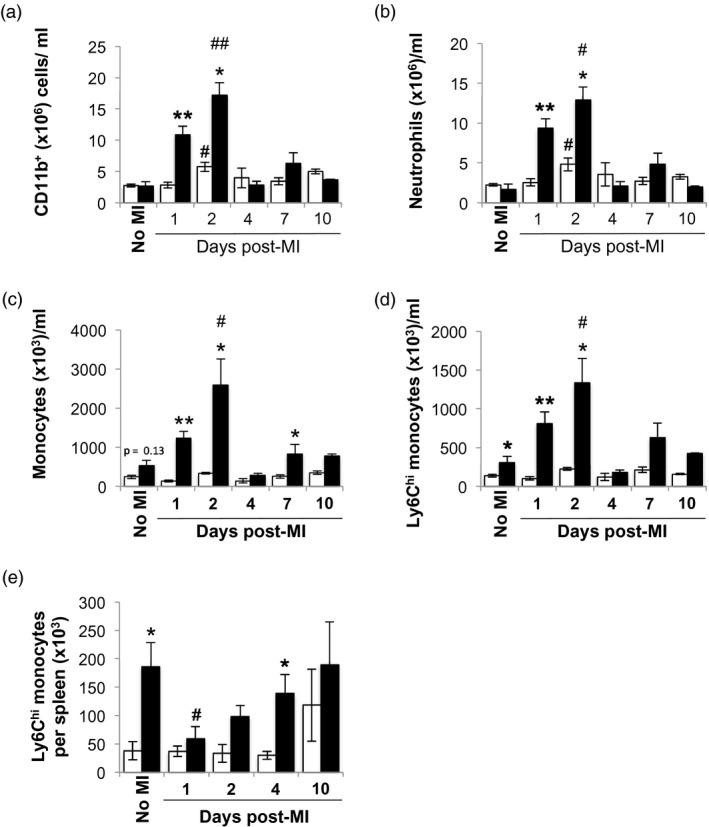
Enhanced peripheral blood monocytosis and mobilization of splenic monocytes in C57BL/6 mice compared with BALB/c mice following myocardial infarction (MI). (a) Peripheral blood CD11b^+^ cells. (b) Peripheral blood neutrophil count. (c) Peripheral blood monocyte count. (d) Peripheral blood lymphocyte antigen‐6 (Ly6)C^high^ monocyte count (*n* = 3–11/group). (e) Total number of splenic Ly6C^high^ monocytes (*n* = 3–6/group). White bars: wild‐type (WT) BALB/c mice. Black bars: C57BL/6 mice. ^*,#^ *P* < 0·05; ^**, ##^ *P* < 0·01; ^*,**^BALB/c *versus* C57BL/6; ^#,##^no MI *versus* time‐point.

In the peripheral blood at baseline, C57BL/6 mice and BALB/c mice had similar numbers of total myeloid cells, including total monocyte and neutrophil counts (Fig. 2a–c). However, even under steady‐state conditions, blood levels of Ly6C^high^ monocytes in C57BL/6 mice were elevated compared to BALB/c mice (Fig. 2d).

Following MI, total peripheral blood myeloid cell counts peaked at day 2 in both C57BL/6 and BALB/c mice (Fig. 2a). This was due to both a relative neutrophil (Fig. 2b) and Ly6C^high^ monocytosis (Fig. 2d). However, during the acute inflammatory phase (from days 1 to 4), the numbers of peripheral blood neutrophils (Fig. 2b), total monocytes (Fig. 2c), and particularly Ly6C^high^ monocytes (Fig 2d), were lower in BALB/c mice compared with C57BL/6 mice.

Mobilization of splenic Ly6C^high^ monocytes has been described as a key part of the inflammatory phase of infarct healing 3, 23. Similar to the peripheral blood pool, the splenic pool of Ly6C^high^ monocytes in naive C57BL/6 mice was also larger in comparison to BALB/c mice (Fig. 2e). Consistent with these observations, there was a reduction in the number of splenic Ly6C^high^ monocytes at day 1 post‐MI in C57BL/6 mice (Fig. 2e). The splenic monocyte reservoir in these mice was replenished by day 4 post‐MI. In contrast, the numbers of splenic Ly6C^high^ monocytes in BALB/c mice at day 1 post‐MI were not significantly different from naive animals (day 0: 3·8 ± 1·5 × 10^4 ^cell/ml *versus* day 1 post‐MI: 3·7 ± 0·9 × 10^4 ^cell/ml, *P* = 0·8; Fig. 2e). This would suggest that splenic monocyte mobilization following MI is not as prominent in BALB/c mice.

### Myeloid cell recruitment to the infarct zone in C57BL/6 mice is enhanced and more persistent than in BALB/c mice

**Figure 3:**
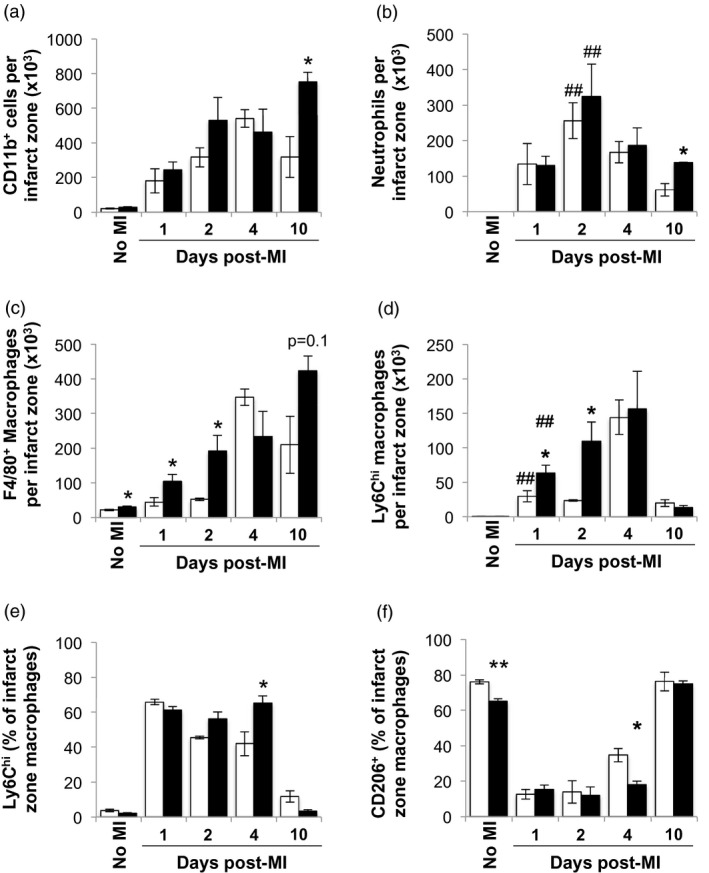
Earlier recruitment and delayed transition of infarct zone macrophages towards the anti‐inflammatory macrophage phenotype in C57BL/6 mice. (a) Total number of CD45^+^CD11b^+^ cells in the infarct zone. (b) Total number of lymphocyte antigen‐6 (Ly6)G^+^ neutrophils in the infarct zone. (c) Total number of F4/80^+^ macrophages in the infarct zone. (d) Total number of Ly6C^high^ macrophages in the infarct zone. (e) Proportion of Ly6C^high^ expressing macrophages in the infarct zone. (f) Proportion of CD206 expressing macrophages in the infarct zone (*n* = 3–6/group). White bars: wild‐type (WT) BALB/c mice. Black bars: C57BL/6. ^*^ *P* < 0·05; ^**, ##^ *P* < 0·01; ^*,**^BALB/c *versus* C57BL/6; ^##^no MI *versus* time‐point.

To investigate whether the higher peripheral blood count of proinflammatory Ly6C^high^ monocytes in C57BL/6 mice was associated with increased and prolonged inflammation, the myeloid cell infiltrate of the infarct zone was characterized (Fig. 3a).

Neutrophils are among the first innate immune cell recruited to the infarcted myocardium 3. In both C57BL/6 and BALB/c mice, the inflammatory infiltrate following MI was dominated by neutrophils in the first 48 h. Unlike in the blood, similar numbers of neutrophils were found in the infarct zones of both mouse strains during the early inflammatory phase (Fig. 3b). Thereafter, the number of neutrophils declined within the infarct zones of both C57BL/6 and BALB/c mice. However, by day 10 post‐MI, the remaining number of neutrophils in the infarct zone was higher in C57BL/6 mice compared to BALB/c mice, indicating a prolonged inflammatory response (Fig. 3b).

In the steady state, the numbers of F4/80^+^ macrophages in the myocardium were higher in C57BL/6 mice compared to BALB/c mice (Fig. 3c). Following MI, macrophages accumulated in the infarct zone in both BALB/c and C57BL/6 mice (Fig. 3c). In the early phase of MI, these were largely comprised of proinflammatory F4/80^+^Ly6C^high^ monocyte‐derived macrophages (Fig. 3d). In line with enhanced blood monocytosis, C57BL/6 mice showed initially greater recruitment of Ly6C^high^ monocyte‐derived macrophages to the infarcted myocardium at days 1 and 2 post‐MI compared to BALB/c mice (Fig. 3d). At day 4 post‐MI equal numbers of proinflammatory, Ly6C^high^ and total macrophages were found in both strains, indicating similar degrees of peak inflammation. Thereafter, total infarct zone macrophage counts declined in BALB/c mice, whereas they further increased in C57BL/6 mice at day 10 post‐MI (day 4: 5·7 ± 0·5 × 10^4^ cells *versus* day 10 9·5 ± 1·5 × 10^4 ^cells; *P* = 0·006) in keeping with a prolonged inflammatory response (Fig. 3c).

### C57BL/6 mice display persistence of inflammatory monocyte‐derived macrophages and delayed acquisition of an anti‐inflammatory macrophage phenotype following MI

In both mouse strains, at day 1 post‐MI the majority of F4/80^+^ macrophages in the infarct zone were Ly6C^high^ (Fig. 3e), indicative of their monocyte origin 23, 24. The resolution phase of infarct healing is marked by the transition of Ly6C^high^ monocyte‐derived macrophages towards a reparative Ly6C^low^, CD206^+^ phenotype 24. Accordingly, the proportion of Ly6C^high^ monocyte‐derived macrophages declined in BALB/c mice over time (proportion of Ly6C^high^ monocyte‐derived macrophages in BALB/c mice, day 1 post‐MI: 65·8 ± 1·5% *versus* day 4 post‐MI: 41·9 ± 4%; *P* = 0·01), whereas C57BL/6 mice maintained a high percentage of Ly6C^high^ monocyte‐derived macrophages up to day 4 post‐MI [proportion of Ly6C^high^ monocyte‐derived macrophages in C57BL/6 mice, day 1 post‐MI: 61·2 ± 2·0% *versus* day 4 post‐MI: 65·2 ± 4%; *P* = not significant (n.s.)].

The vast majority of cardiac macrophages in naive hearts from both mouse strains expressed CD206 (Fig. 3f). However, already in steady state, the proportion of CD206 expressing macrophages was higher in hearts from naive BALB/c mice in comparison to naive C57BL/6 mice (*P* = 0·002). The proportion of infarct zone macrophages expressing CD206 in C57BL/6 mice and BALB/c mice was reduced to below 20% at day 1 post‐MI (Fig. 3f), consistent with the concurrent influx of proinflammatory monocyte‐derived macrophages (Fig. 3d,e). Enhanced transition to CD206‐expressing macrophages was detectable in BALB/c mice as of day 4 post‐MI but not in C57BL/6 mice (Fig. 3f). By day 10 post‐MI, CD206 expression on infarct zone macrophages had returned to baseline in both BALB/c and C57BL/6 mice (Fig. 3f).

### C57BL/6 mice show an enhanced inflammatory response in the remote myocardium and pericardial adipose tissue

**Figure 4:**
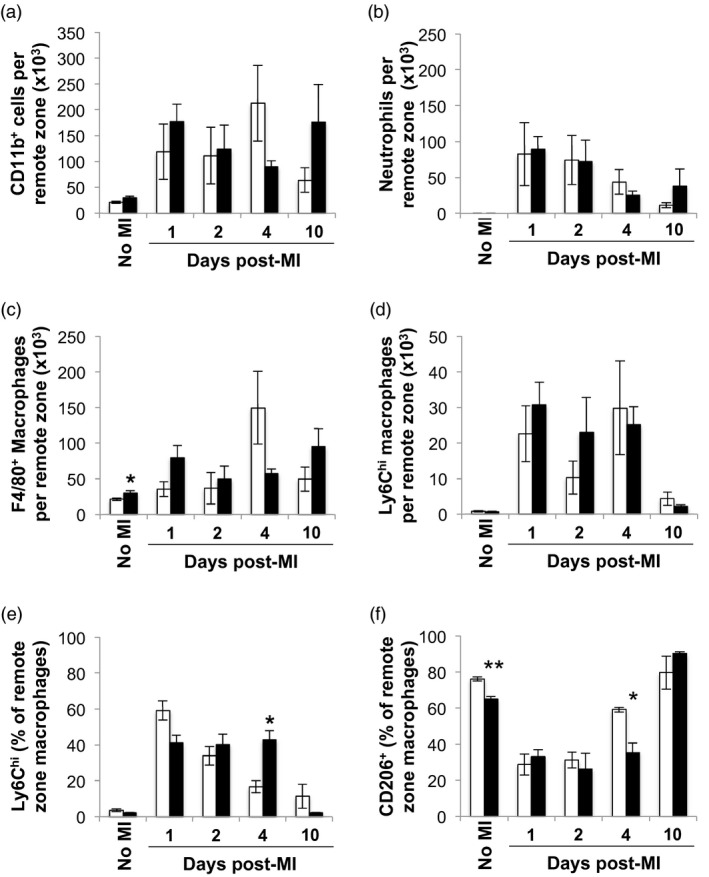
Delayed transition of C57BL/6 remote zone macrophages towards the anti‐inflammatory macrophage phenotype. (a) Total number of CD11b^+^ cells in the remote zone. (b) Total number of neutrophils in the remote zone. (c) Total number of F4/80^+^ macrophages in the remote zone. (d) Total number of lymphocyte antigen‐6 (Ly6)C^high^ macrophages in the remote zone. (e) Proportion of Ly6C^high^ expressing macrophages in the remote zone. (f) Proportion of CD206 expressing macrophages in the remote zone (*n* = 3–6/group). White bars: wild‐type (WT) BALB/c mice. Black bars: C57BL/6. ^*,#^ *P < *0·05; ^**^ *P* < 0·01; ^*,**^BALB/c *versus* C57BL/6; ^#^no MI *versus* time‐point.

The infarct zone displays a more pronounced accumulation of monocyte‐derived macrophage infiltration following MI than the remote non‐infarcted zone 25. However, the relationship between immune cell infiltration of the remote zone and cardiac repair during the acute phase has not previously been explored. Surprisingly, unlike the infarct zone, both mouse strains showed similar degrees of total and Ly6C^high^ macrophage infiltration in remote zones of the heart at days 1 and 2 post‐MI (Fig. 4a–d). However, in keeping with data from the infarct zone, the proportion of Ly6C^hi^ monocyte‐derived macrophages in the remote zone at day 4 post‐MI was higher in C57BL/6 mice compared to BALB/c mice (*P* = 0·04; Fig. 4e). In addition, as observed in the infarct zone, the transition to a CD206^+^ anti‐inflammatory macrophage phenotype in the remote zone was delayed in C57BL/6 mice compared with BALB/c mice (Fig. 4f).

**Figure 5:**
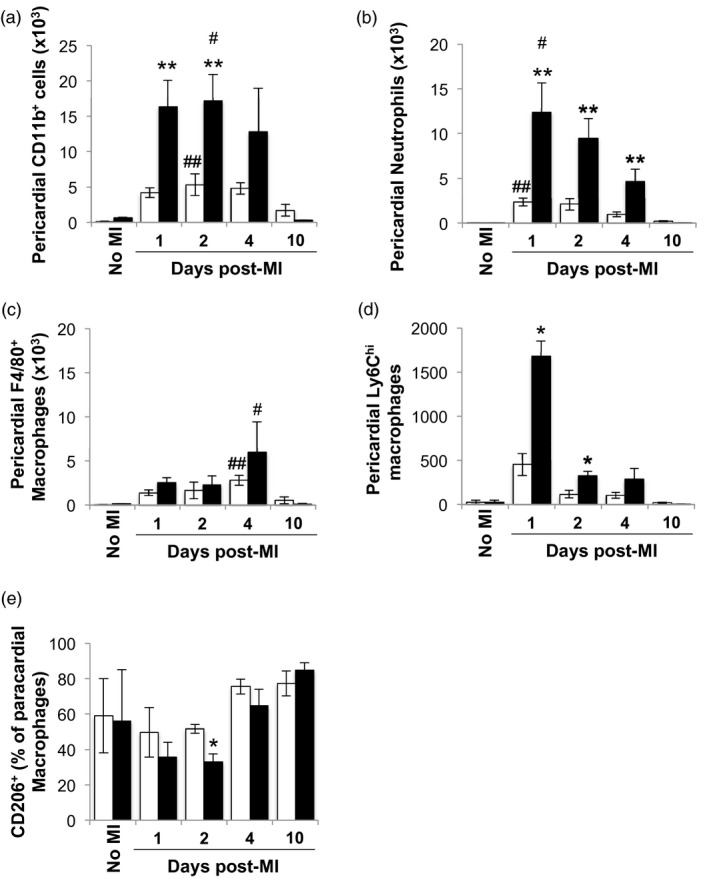
Delayed transition towards the anti‐inflammatory macrophage phenotype of C57BL/6 macrophages recruited to the pericardial adipose tissue following myocardial infarction (MI). (a) Total number of CD11b^+^ cells in the pericardial adipose tissue. (b) Total number of neutrophils in the pericardial adipose tissue. (c) Total number of F4/80^+^ macrophages in the pericardial adipose tissue. (d) Total number of lymphocyte antigen‐6 (Ly6)C^high^ macrophages in the pericardial adipose tissue. (e) Proportion of CD206 expressing macrophages in the paracardial adipose tissue (*n* = 3–6/group). White bars: wild‐type (WT) BALB/c mice. Black bars: C57BL/6. ^*,#^ *P* < 0·05; ^**, ##^ *P* < 0·01; ^*,**^BALB/c *versus* C57BL/6; ^#, ##^no MI *versus* time‐point.

Recently it has been demonstrated that pericardial adipose tissue regulates the innate immune response in the infarcted myocardium 26. Similar to the infarct zone, neutrophils were the predominant myeloid cell type infiltrating the pericardial adipose tissue of both C57BL/6 and BALB/c mice during the early inflammatory phase, but peaked earlier at day 1 post‐MI (Fig. 5a,b). In contrast to the infarct zone, the total numbers of pericardial adipose tissue neutrophils were significantly higher in C57BL/6 mice compared to BALB/c mice until day 4 post‐MI (Fig. 5b). In keeping with the infarct zone, during the early inflammatory phase the representation of Ly6C^high^ monocyte‐derived macrophages was higher in the pericardial adipose of C57BL/6 compared to BALB/c mice (Fig. 5d). Furthermore, the transition of macrophages from a proinflammatory to a proresolution phenotype within the pericardial adipose tissue of C57BL/6 mice preceded that of infarct zone macrophages (Fig. 5e). Macrophage numbers in the pericardial adipose tissue of both strains returned to baseline by day 10 post‐MI (Fig. 5c), which was before the infarct zone.

## Discussion

Little is known about mouse strain differences in myeloid cell recruitment and resolution of the innate immune response following MI. This study highlights the local and systemic proinflammatory bias of C57BL/6 mice compared to BALB/c mice following MI. The systemic inflammatory response in C57BL/6 mice following MI was more pronounced, with greater peripheral blood monocytosis, splenic monocyte mobilization and myeloid cell infiltration of pericardial adipose tissue. This was associated with a faster and more prolonged macrophage accumulation, as well as delayed transition towards an anti‐inflammatory macrophage phenotype in the infarct zone of C57BL/6 mice. Prolonged inflammation resulted in impaired infarct healing with an increased susceptibility of C57BL/6 mice to cardiac rupture.

Ly6C^–^ cardiac‐resident macrophages in intact adult mouse hearts are replaced by day 1 following myocardial infarction 21, 27 with macrophages derived from Ly6C^high^ bone marrow derived monocytes 3, 27, 28. Swirski *et al.* showed that in mice on the C57BL/6 background, up to 75% of monocytes acutely recruited to the infarcted myocardium originate from the splenic monocyte reservoir 23. In the current study, the reduction in splenic Ly6C^high^ monocyte numbers early post‐MI in C57BL/6 mice supports this conclusion. In contrast, splenic Ly6C^high^ monocyte numbers were unchanged in BALB/c mice following MI, despite Ly6C^high^ monocyte‐derived macrophage accumulation in the infarct zone. These findings suggest that BALB/c mice do not have as prominent a recruitment of splenic monocytes to the infarct zone and may rely more on bone marrow‐derived monocytes. Recent data in a nematode infection model also revealed profound differences between BALB/c and C57BL/6 mice in terms of myeloid cell dynamics 29. Monocyte recruitment from the bone marrow was greater in the BALB/c strain, and the bone marrow monocyte‐derived macrophages adopted an immune‐suppressive phenotype on recruitment to the site of infection 29. Conversely, Ly6C^high^ monocyte mobilisation from the spleen following MI has been shown to enhance inflammation and proteolytic activity in the infarct zone 4. Thus, differential mobilization of splenic versus bone marrow‐derived monocytes following MI in C57BL/6 and BALB/c mice, respectively, may underlie the more pronounced inflammatory response seen in the infarct zone of C57BL/6 mice.

It is well established that macrophages have a key role in infarct healing in both C57BL/6 and BALB/c mice 3. This is demonstrated by impaired removal of necrotic or damaged tissue and a dramatic increase in the rate of cardiac rupture when monocytes and macrophages are depleted with clodronate liposome injection following MI 30. However, when recruitment of proinflammatory Ly6C^high^ monocyte‐derived macrophages is enhanced during the early inflammatory phase, this also leads to impaired infarct healing 3, 4. Accordingly, in this study, C57BL/6 mice with their higher cardiac rupture rate displayed greater peripheral blood Ly6C^high^ monocytosis following MI. In the infarct zone, this was associated with an increased early accumulation of Ly6C^high^ monocyte‐derived macrophages, as well as a prolonged accumulation of macrophages post‐MI. These results emphasize the fine balance between detrimental and beneficial effects of monocyte recruitment during infarct healing. However, it is currently unclear whether the enhanced influx of proinflammatory Ly6C^high^ monocytes results in further tissue degradation or limits otherwise protective, reparative processes. Increased recruitment of proinflammatory Ly6C^high^ monocytes to the infarct zone has been associated with increased expression of matrix metalloproteinase (MMP)‐2 and ‐9 on immunohistochemistry of infarct tissue 4. The gelatinase group of MMPs, which includes MMP‐2 and MMP‐9, cleave soluble fragments of collagen and promote the development of infarct rupture 31. van den Borne *et al.* 9 have previously reported no difference in levels of either active or latent MMP‐2 and ‐9 in infarct tissue collected at day 3 post‐MI from BALB/c and C57BL/6 mice; they also found no difference in the extent of inflammatory cell detection by histological staining. However, cardiac rupture did not occur until later than 3 days in that study. Flow cytometric evaluation utilized in the current study offers greater myeloid cell discrimination and comprehensive quantitative assessment of the inflammatory infiltrate in the infarcted myocardium. *In‐vitro* data indicate that macrophages from C57BL/6 mice intrinsically have a proinflammatory bias compared to BALB/c macrophages, as they produce more tumour necrosis factor (TNF)‐α and IL‐12 in response to stimulation with lipopolysaccharide 19. Macrophages from BALB/c mice also facilitate wound healing by more readily metabolizing arginine to ornithine 14, which is a precursor for collagen synthesis 32. Thus, in addition to the differences in cellular recruitment and source discussed above, the inherent difference in responsiveness of macrophages in C57BL/6 and BALB/c mice may also contribute to inadequate extracellular matrix deposition, which may account for the longer scar size and increased susceptibility of C57BL/6 mice for cardiac rupture. There are also differences in T cell populations between mouse strains, such that BALB/c mice show a higher CD4^+^ to CD8^+^ T cell ratio in lymphoid organs than C57BL/6 mice 33. Moreover, CD4^+^ T cells from C57BL/6 mice produce lower levels of IL‐2 than BALB/c mice 34, 35. Therefore, in addition to the differences in myeloid cell recruitment described herein, differences in the number of CD4^+^ T cells present in a mouse strain may influence Th1/Th2 polarization and potentially cardiac remodelling post‐MI 36.

Horckman *et al.* have recently shown that removal of pericardial adipose tissue is associated with an almost 50% reduction in recruitment of neutrophils to the myocardium following MI 26. Findings from the present study, showing that peak neutrophil infiltration of pericardial adipose tissue precedes the peak of infarct zone neutrophil count, supports the involvement of pericardial adipose tissue in regulating the infarct zone inflammatory response. In particular, higher neutrophil accumulation in pericardial adipose tissue of C57BL/6 mice was associated with increased infarct zone neutrophil accumulation at day 10 post‐MI. Total macrophage count and the transition of macrophages from a proinflammatory to a proresolution phenotype within the pericardial adipose tissue of C57BL/6 mice also preceded that of infarct zone macrophages. These findings raise the highly interesting question of whether pericardial adipose tissue modulates the infarct zone immune response, including the resolution of inflammation.

Frequency of specific alleles for TNF‐α, IL‐1β and Toll‐like receptor 4 are associated with incidence and severity of clinical inflammatory disease 37, 38, 39, 40. As the post‐MI inflammatory response has an important influence on cardiac remodelling 41, differential distribution of polymorphisms in genes encoding components of inflammatory pathways are highly likely to contribute to variation in susceptibility to adverse remodelling in the human population. Immunosenescence of the innate immune response is also associated with impaired wound healing in the elderly population, and is characterized by sustained elevation of proinflammatory cytokines such as IL‐6 and TNF‐α 42. Identifying the genetic profile that predicts the inflammatory response following MI would help to identify those most at risk of adverse outcomes and open up the potential for targeted therapy to limit detrimental cardiac remodelling.

Current medical therapy for cardiovascular risk reduction, such as aspirin 43, statins 44 and angiotensin‐converting‐enzyme (ACE) inhibitors 45, also have anti‐inflammatory effects which probably modulate the immune response following MI. Interestingly, ACE inhibitors have a pleiotropic anti‐inflammatory effect by reducing both monocyte mobilization from the spleen and monocyte recruitment to the infarcted myocardium 45. The outcome of the present study would support intervention with, for example, IL‐4 and IL‐10 in MI patient subgroups, to modulate the anti‐inflammatory phenotype of macrophages during repair after MI to beneficially influence long‐term outcome.

Elevated peripheral blood Ly6C^high^ monocyte count in C57BL/6 mice was associated with a more pronounced and prolonged inflammatory response within the infarct zone and a lower survival rate, compared to BALB/c mice. Differences in systemic and local inflammation between C57BL/6 and BALB/c mice highlight the therapeutic potential of modulating resolution of the innate immune response following MI for the benefit of successful infarct healing. Considering the pronounced involvement of pericardial adipose tissue and the splenic monocyte reservoir in the inflammatory response following MI, these tissues may be important therapeutic targets in order to facilitate successful infarct healing.

## Conflict of interests

The authors declare no competing interests.

## Author contributions

I. S. T., G. A. G., J. E. A. and D. R. designed the studies; I. S. T., A. T. and I. M. conducted experiments, I. S. T., A. T. and I. M. acquired data, I. S. T. analysed data, G. A. G., J. E. A. and A.G. R. provided reagents and I. S. T., G. A. G., J. E. A., D. E. N., A. G. R., D. R. and I. M. contributed to writing of the manuscript.

## Supporting information


**Fig. S1.** Representative flow cytometry plots showing gating strategy to identify neutrophils (NΦ), total macrophages (MΦ) and Ly6Chi macrophages (Ly6C^hi^ MΦ).

**Table S1.** Cardiac function at Day 7 following MI in WT BALB/c and C57BL/6 mice (*n* = 7‐10 /group).

**Table S2.** Antibodies for Flow cytometry.
