# Supplementary material for: Enhanced monocyte recruitment and delayed alternative macrophage polarization accompanies impaired repair following myocardial infarction in C57BL/6 compared to BALB/c mice
Source: Clin Exp Immunol. 2019 Jun 17;198(1):83–93. doi: 10.1111/cei.13330 (PMC6718279; doi:10.1111/cei.13330)
Supplement: Supplementary file 2 — Table S1. Cardiac function at Day 7 following MI in WT BALB/c and C57BL/6 mice (n = 7‐10 /group). [file CEI-198-83-s002.docx]

|  | BALB/c  (n=10) | C57BL/6  (n=7) | p-value |
| --- | --- | --- | --- |
| End-Diastolic Area (mm^2^) | 33.1±1.5 | 32.0±2.1 | 0.48 |
| Fractional Area Change (%) | 12.5±2.2 | 17.6±2.6 | 0.16 |

**Supplementary Table S1:** Cardiac function at Day 7 following MI in WT BALB/c and C57BL/6 mice (n=7-10 /group).
