# Supplementary material for: Enhanced monocyte recruitment and delayed alternative macrophage polarization accompanies impaired repair following myocardial infarction in C57BL/6 compared to BALB/c mice
Source: Clin Exp Immunol. 2019 Jun 17;198(1):83–93. doi: 10.1111/cei.13330 (PMC6718279; doi:10.1111/cei.13330)
Supplement: Supplementary file 3 — Table S2. Antibodies for Flow cytometry. [file CEI-198-83-s003.docx]

| Monoclonal rat anti-mouse antibody | Clone | Fluorophores | Manufacture | Concentration |
| --- | --- | --- | --- | --- |
| CD45.2 | 104 | PE Cy7 | Biolegend | 1:100 |
| CD45.2 | 104 | BV650 | Biolegend | 1:500 |
| CD11b | M1/70 | AF700 | Biolegend | 1:200 |
| F4/80 | BM8 | PE Cy7 | BD Biosciences | 1:200 |
| Ly6G | 1A8 | Pacific Blue | Biolegend | 1:200 |
| CD206 | C068C2 | FITC | Biolegend | 1:100 |
| Ly6C | HK1-4 | PerCP Cy5.5 | BD Pharmingen | 1:100 |
| CD115 | T38-320 | APC | BD Pharmingen | 1:100 |
|  |  | DAPI | Life Technologies | 1:1000 |
| anti-CD16/32 | 2.4G2 |  | BD Bioscience | 1:200 |

**Supplementary Table S2:** Antibodies for Flow cytometry
