# Supplementary material for: Enhanced monocyte recruitment and delayed alternative macrophage polarization accompanies impaired repair following myocardial infarction in C57BL/6 compared to BALB/c mice
Source: Clin Exp Immunol. 2019 Jun 17;198(1):83–93. doi: 10.1111/cei.13330 (PMC6718279; doi:10.1111/cei.13330)
Supplement: Supplementary file 4 [file CEI-198-83-s004.docx]

**Single Cell preparation for flow cytometry**

30 μL tail blood samples (in 30 μL 3.2% citrate buffer) were stained with a mixture of antibodies (Supplementary Table S2 online) at 4°C for 30 minutes. Subsequently, tail blood samples underwent red blood cell lysis with FACS lysing buffer (BD Bioscience, 1:10 dilution in dH2O) and were then washed PBS^-/-^. Total white blood cell numbers were determined using Flow-Check™ fluorospheres (Life Technology).

Spleens were collected from naïve mice and following MI, and prepared as a single-cell suspension by mechanical dissociation and triturated through a 40 μm nylon mesh. The cell suspensions were centrifuged, treated with red blood cell lysing buffer (Sigma) and washed with PBS^-/-^ and total leukocyte numbers were determined. Hearts were perfused with heparinized saline and the left ventricles harvested from naïve mice and following MI. The infarcted left ventricle was dissected in order to separate it into infarct and remote zone myocardium.

Heart samples were transferred to gentle MACS™ tubes and pericardial adipose tissue transferred to 1.5 ml eppendorf tubes containing Hanks’ balanced salt solution (HBSS) with Ca^2+^ and Mg^2+^ in which was dissolved collagenase D (1.25 mg/mL) (Roche, Lakewood, NJ) and DNase I (60U U/mL) (Sigma, St. Louis, MO). Infarct and remote zone myocardium samples were homogenized using Heart protocol 1 on a MACS™ Dissociator. Single-cell suspensions of heart tissue and pericardial adipose tissue samples were prepared by incubating the enzyme mix at 37°C for 30 minutes whilst being gently agitated. The heart tissues samples underwent another round of mechanical dissociation using Heart protocol 2 on a MACS™ Dissociator. The heart tissue and pericardial adipose tissue cell suspensions were then triturated through a 40 μm nylon mesh, and washed with PBS^-/-^ and total leukocyte numbers were determined.

Single cell suspensions (<1x10^6^) from the heart, pericardial adipose, and spleen of animals were incubated with a mixture of fluorophore-conjugated antibodies (Online Table 12) at 4°C for 30 minutes and then washed with PBS^-/-^. DAPI (1 μL/mL) was added to the samples for fluorescence associated cell sorting, immediately prior to acquisition.

**Flow cytometry**

Flow cytometric analysis was performed on an LSR II instrument (BD Biosciences) and analyzed using FlowJo software (Tree Star).

Results for the heart digests are expressed as cell number per infarct zone or remote zone, and total counts were calculated for spleens and the peripheral blood. Live single cells were gated for by excluding dead cells using DAPI, followed by singlet gates and subsequently by granularity and size. Fluorochrome conjugated antibodies were used to define cell populations of interest (Supplementary Fig. S1 online).
